# Crystal structure of *N*-carbamo­thioyl-2-methyl­benzamide

**DOI:** 10.1107/S2056989015009585

**Published:** 2015-05-28

**Authors:** Farook Adam, Nadiah Ameram, Wai Mun Tan

**Affiliations:** a School of Chemical Sciences, Universiti Sains Malaysia, 11800 Georgetown, Penang, Malaysia

**Keywords:** crystal structure, benzamide, thio­urea, hydrogen bonding

## Abstract

There are two mol­ecules in the asymmetric unit of the title compound, C_9_H_10_N_2_OS. In one, the dihedral angle between the aromatic ring and the carbamo­thioyl group is 52.31 (7)° and in the other it is 36.16 (6)°. Each mol­ecule features an intra­molecular N—H⋯O hydrogen bond, which generates an *S*(6) ring and the O and S atoms have an *anti* disposition. In the crystal, mol­ecules are linked by N—H⋯S and N—H⋯O hydrogen bonds, generating separate [130] and [1-30] infinite chains. Weak C—H⋯O and C—H⋯S inter­actions are also observed.

## Related literature   

For related structures, see: Saeed & Flörke (2007 ▸); Shoukat *et al.* (2007 ▸); Hassan *et al.* (2008*a* ▸,*b* ▸,*c* ▸); Ameram *et al.* (2015 ▸).
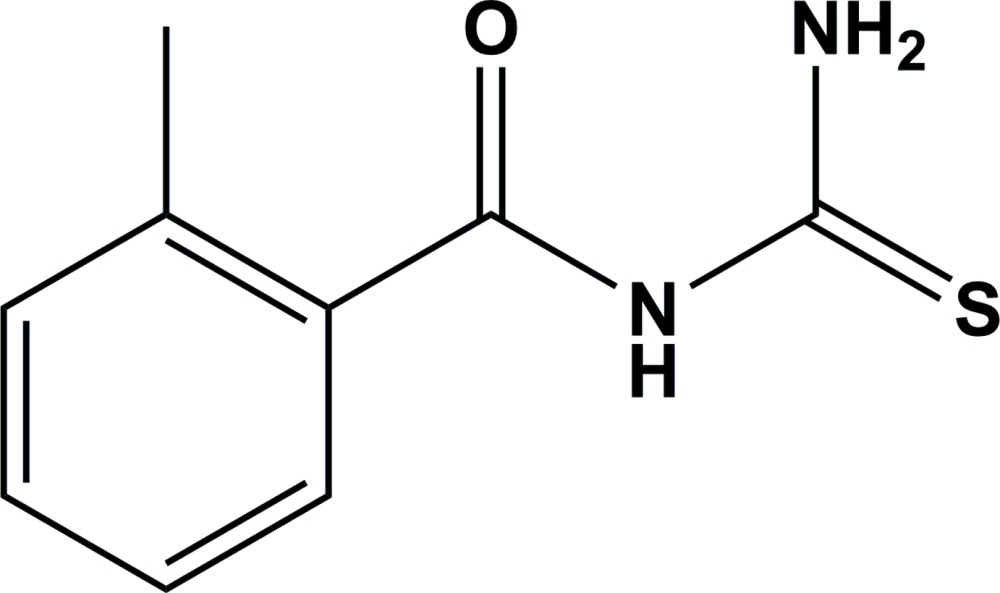


## Experimental   

### Crystal data  

C_9_H_10_N_2_OS
*M* *_r_* = 194.25
Monoclinic, 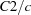

*a* = 22.7886 (12) Å
*b* = 7.1133 (3) Å
*c* = 25.5388 (13) Å
β = 113.664 (3)°
*V* = 3791.8 (3) Å^3^
*Z* = 16
Mo *K*α radiation
μ = 0.30 mm^−1^
*T* = 100 K
0.46 × 0.33 × 0.10 mm

### Data collection  

Bruker APEX DUO CCD diffractometer
Absorption correction: multi-scan (*SADABS*; Bruker, 2009 ▸) *T*_min_ = 0.814, *T*_max_ = 0.872
70711 measured reflections
5697 independent reflections
4862 reflections with *I* > 2σ(*I*)
*R*_int_ = 0.051

### Refinement  

*R*[*F* ^2^ > 2σ(*F* ^2^)] = 0.043
*wR*(*F* ^2^) = 0.110
*S* = 1.10
5697 reflections
261 parameters
H atoms treated by a mixture of independent and constrained refinement
Δρ_max_ = 0.45 e Å^−3^
Δρ_min_ = −0.22 e Å^−3^

### 

Data collection: *APEX2* (Bruker, 2009 ▸); cell refinement: *SAINT* (Bruker, 2009 ▸); data reduction: *SAINT*; program(s) used to solve structure: *SHELXS2013* (Sheldrick, 2008 ▸); program(s) used to refine structure: *SHELXL2014* (Sheldrick, 2015 ▸); molecular graphics: *SHELXTL* (Sheldrick, 2008 ▸); software used to prepare material for publication: *PLATON* (Spek, 2009 ▸).

## Supplementary Material

Crystal structure: contains datablock(s) I, New_Global_Publ_Block. DOI: 10.1107/S2056989015009585/hb7426sup1.cif

Structure factors: contains datablock(s) I. DOI: 10.1107/S2056989015009585/hb7426Isup2.hkl

Supporting information file. DOI: 10.1107/S2056989015009585/hb7426Isup3.cml

**Fig. 1.**
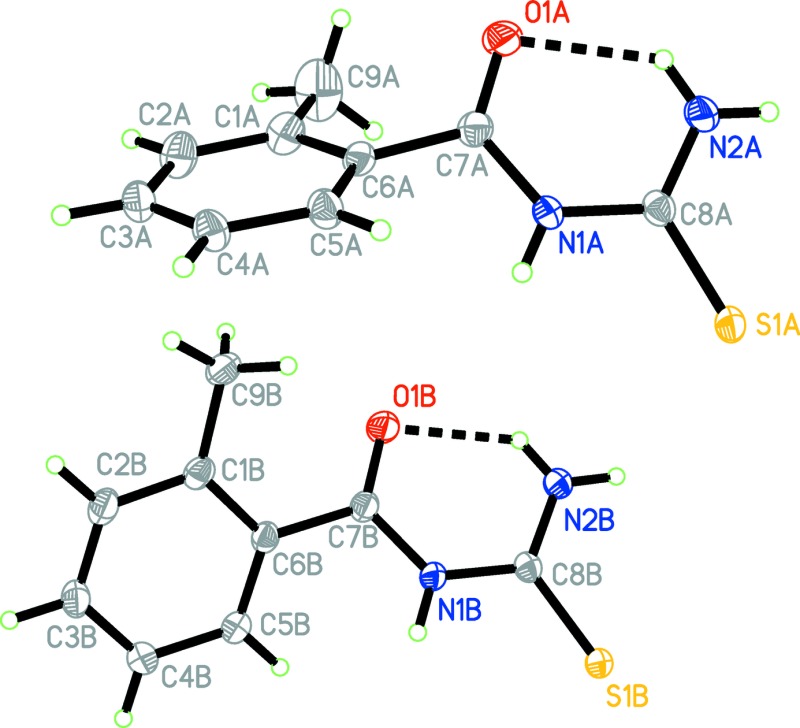
A view of the molecular structure of the title compound, showing the atom labellling. Displacement ellipsoids are drawn at the 50% probability level.

. DOI: 10.1107/S2056989015009585/hb7426fig1.tif
A view of the mol­ecular structure of the title compound, showing the atom labellling. Displacement ellipsoids are drawn at the 50% probability level.

**Fig. 2.**
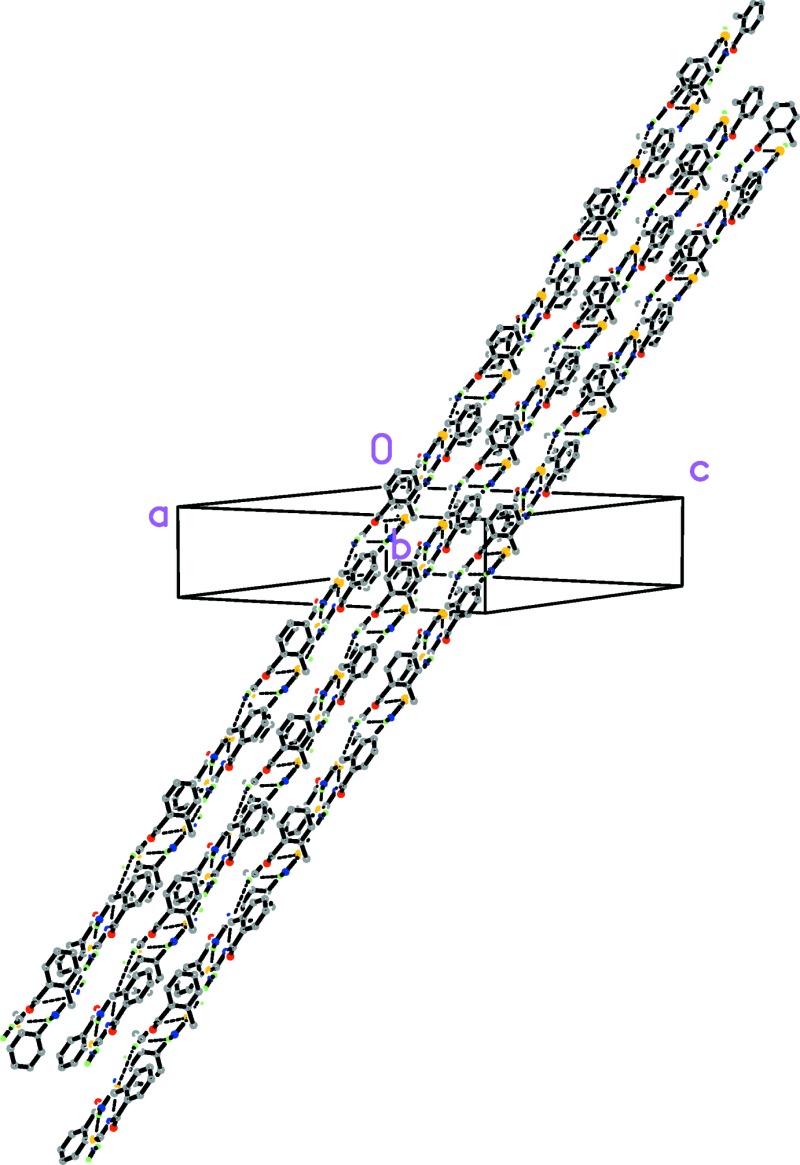
A view of the crystal packing of the title compound. Hydrogen bonds are shown as dashed lines (see Table 1 for details).

. DOI: 10.1107/S2056989015009585/hb7426fig2.tif
A view of the crystal packing of the title compound. Hydrogen bonds are shown as dashed lines (see Table 1 for details).

CCDC reference: 1401733

Additional supporting information:  crystallographic information; 3D view; checkCIF report

## Figures and Tables

**Table 1:** Hydrogen-bond geometry (, )

*D*H*A*	*D*H	H*A*	*D* *A*	*D*H*A*
N1*A*H1*A*S1*A* ^i^	0.80(2)	2.60(2)	3.3227(16)	151.2(18)
N1*B*H1*B*S1*B* ^ii^	0.84(2)	2.65(2)	3.4780(14)	172(2)
N2*A*H2*A*S1*B* ^iii^	0.85(2)	2.49(2)	3.2945(15)	157.8(19)
N2*B*H2*B*O1*B*	0.83(2)	1.98(2)	2.6404(18)	136(2)
N2*A*H3*A*O1*A*	0.83(2)	2.02(2)	2.6515(19)	133(2)
N2*B*H3*B*S1*A* ^iii^	0.89(2)	2.49(2)	3.3800(14)	177(2)
C5*B*H5*BA*O1*A* ^iv^	0.95	2.45	3.3584(19)	160
C9*B*H9*BA*S1*A* ^i^	0.98	2.80	3.6946(17)	152

Symmetry codes: (i) 
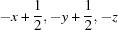
; (ii) 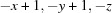; (iii) 
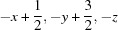
; (iv) 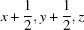.

## supplementary crystallographic information

### S1. Introduction

In the crystal, molecules are linked by pairs of C=O—H hydrogen bonds with the methyl group from molecule (B) is facing the group from the molecule (A) forming slabs which is parallel to the benzene ring plane (A) as the bond of C6–C7 (A and B) can free to rotate.

### S2. Experimental

The title compound (Fig. 1) is a benzoyl thio­urea inter­mediate to a compound recently reported by us (Ameram *et al.*, 2015) and there is no substituent at the end of thio­amide group.

### S2.1. Synthesis and crystallization

Freshly prepared substituted *o*-benzoyl chloride (13 mmol) was added dropwise to a stirred acetone solution (30 ml) of ammonium thio­cyanate (13 mmol). The mixture was stirred for 10 min. A white side product which is ammonium chloride was filtered off. The compound was left at room temperature to crystallize, to yield colourless plates of the title compound.

### S2.2. Refinement

Crystal data, data collection and structure refinement details are summarized in Table 2. The H-atoms on the N atoms were located in a difference-Fourier map and were freely refined. All other H atoms were positioned geometrically and refined using a riding model with C—H = 0.93–0.96 Å and *U*_iso_(H) = 1.2*U*_eq_(aromatic C) or 1.5*U*_eq_(methyl C).

### Crystal data

**Table:**

C_9_H_10_N_2_OS	*F*(000) = 1632
*M**_r_* = 194.25	*D*_x_ = 1.361 Mg m^−^^3^
Monoclinic, *C*2/*c*	Mo *K*α radiation, λ = 0.71073 Å
*a* = 22.7886 (12) Å	Cell parameters from 9948 reflections
*b* = 7.1133 (3) Å	θ = 3.0–29.9°
*c* = 25.5388 (13) Å	µ = 0.30 mm^−^^1^
β = 113.664 (3)°	*T* = 100 K
*V* = 3791.8 (3) Å^3^	Plate, colourless
*Z* = 16	0.46 × 0.33 × 0.10 mm

### Data collection

**Table:**

Bruker APEX DUO CCD diffractometer	5697 independent reflections
Radiation source: fine-focus sealed tube	4862 reflections with *I* > 2σ(*I*)
Graphite monochromator	*R*_int_ = 0.051
φ and ω scans	θ_max_ = 30.4°, θ_min_ = 1.7°
Absorption correction: multi-scan (*SADABS*; Bruker, 2009)	*h* = −32→32
*T*_min_ = 0.814, *T*_max_ = 0.872	*k* = −10→10
70711 measured reflections	*l* = −36→36

### Refinement

**Table:**

Refinement on *F*^2^	0 restraints
Least-squares matrix: full	Hydrogen site location: mixed
*R*[*F*^2^ > 2σ(*F*^2^)] = 0.043	H atoms treated by a mixture of independent and constrained refinement
*wR*(*F*^2^) = 0.110	*w* = 1/[σ^2^(*F*_o_^2^) + (0.0488*P*)^2^ + 4.2106*P*] where *P* = (*F*_o_^2^ + 2*F*_c_^2^)/3
*S* = 1.10	(Δ/σ)_max_ = 0.001
5697 reflections	Δρ_max_ = 0.45 e Å^−^^3^
261 parameters	Δρ_min_ = −0.22 e Å^−^^3^

### Special details

**Table:**

Geometry. All e.s.d.'s (except the e.s.d. in the dihedral angle between two l.s. planes) are estimated using the full covariance matrix. The cell e.s.d.'s are taken into account individually in the estimation of e.s.d.'s in distances, angles and torsion angles; correlations between e.s.d.'s in cell parameters are only used when they are defined by crystal symmetry. An approximate (isotropic) treatment of cell e.s.d.'s is used for estimating e.s.d.'s involving l.s. planes.

### Fractional atomic coordinates and isotropic or equivalent isotropic displacement parameters (Å^2^)

**Table:**

	*x*	*y*	*z*	*U*_iso_*/*U*_eq_	
S1A	0.20453 (2)	0.37036 (5)	0.04623 (2)	0.02123 (9)	
O1A	0.05447 (5)	0.09550 (16)	−0.11126 (5)	0.0285 (3)	
N1A	0.15344 (6)	0.12795 (17)	−0.03834 (5)	0.0195 (2)	
N2A	0.08336 (6)	0.3410 (2)	−0.02644 (7)	0.0264 (3)	
C1A	0.12721 (8)	−0.1174 (2)	−0.16760 (7)	0.0255 (3)	
C2A	0.14877 (8)	−0.2742 (2)	−0.18702 (7)	0.0290 (3)	
H2AA	0.1428	−0.2801	−0.2260	0.035*	
C3A	0.17880 (7)	−0.4224 (2)	−0.15103 (7)	0.0257 (3)	
H3AA	0.1925	−0.5289	−0.1656	0.031*	
C4A	0.18880 (7)	−0.4154 (2)	−0.09401 (7)	0.0239 (3)	
H4AA	0.2096	−0.5165	−0.0692	0.029*	
C5A	0.16820 (7)	−0.2595 (2)	−0.07329 (6)	0.0215 (3)	
H5AA	0.1756	−0.2531	−0.0340	0.026*	
C6A	0.13688 (7)	−0.11301 (19)	−0.10979 (6)	0.0190 (3)	
C7A	0.11026 (7)	0.0452 (2)	−0.08773 (6)	0.0202 (3)	
C8A	0.14215 (7)	0.27880 (19)	−0.00939 (6)	0.0186 (3)	
C9A	0.09650 (12)	0.0421 (3)	−0.20800 (9)	0.0462 (5)	
H9AA	0.1169	0.1608	−0.1906	0.069*	
H9AB	0.0507	0.0472	−0.2159	0.069*	
H9AC	0.1019	0.0222	−0.2438	0.069*	
S1B	0.45007 (2)	0.73146 (5)	−0.00298 (2)	0.02408 (10)	
O1B	0.31413 (5)	0.45230 (16)	−0.16642 (5)	0.0258 (2)	
N1B	0.40643 (6)	0.47799 (17)	−0.08568 (5)	0.0187 (2)	
N2B	0.33660 (6)	0.71847 (19)	−0.08869 (6)	0.0227 (3)	
C1B	0.35431 (7)	0.0654 (2)	−0.17791 (6)	0.0199 (3)	
C2B	0.38290 (8)	−0.0911 (2)	−0.19102 (6)	0.0240 (3)	
H2BA	0.3574	−0.1983	−0.2076	0.029*	
C3B	0.44736 (8)	−0.0943 (2)	−0.18057 (7)	0.0278 (3)	
H3BA	0.4653	−0.2025	−0.1903	0.033*	
C4B	0.48592 (7)	0.0595 (2)	−0.15600 (7)	0.0276 (3)	
H4BA	0.5300	0.0586	−0.1496	0.033*	
C5B	0.45933 (7)	0.2151 (2)	−0.14083 (6)	0.0231 (3)	
H5BA	0.4856	0.3203	−0.1234	0.028*	
C6B	0.39425 (7)	0.2184 (2)	−0.15102 (6)	0.0184 (3)	
C7B	0.36692 (7)	0.3907 (2)	−0.13618 (6)	0.0190 (3)	
C8B	0.39349 (6)	0.64135 (19)	−0.06277 (6)	0.0177 (3)	
C9B	0.28310 (7)	0.0629 (2)	−0.19377 (7)	0.0244 (3)	
H9BA	0.2741	0.1143	−0.1622	0.037*	
H9BB	0.2674	−0.0667	−0.2014	0.037*	
H9BC	0.2615	0.1396	−0.2281	0.037*	
H1B	0.4428 (11)	0.433 (3)	−0.0664 (9)	0.038 (6)*	
H3A	0.0543 (11)	0.291 (3)	−0.0542 (10)	0.039 (6)*	
H1A	0.1904 (10)	0.102 (3)	−0.0300 (8)	0.028 (5)*	
H3B	0.3275 (10)	0.827 (3)	−0.0763 (9)	0.037 (6)*	
H2B	0.3111 (11)	0.667 (3)	−0.1184 (10)	0.039 (6)*	
H2A	0.0765 (10)	0.437 (3)	−0.0096 (9)	0.038 (6)*	

### Atomic displacement parameters (Å^2^)

**Table:**

	*U*^11^	*U*^22^	*U*^33^	*U*^12^	*U*^13^	*U*^23^
S1A	0.02084 (16)	0.02238 (18)	0.01948 (17)	0.00473 (13)	0.00704 (13)	−0.00419 (13)
O1A	0.0193 (5)	0.0211 (5)	0.0368 (6)	0.0039 (4)	0.0025 (4)	−0.0060 (5)
N1A	0.0165 (5)	0.0181 (6)	0.0231 (6)	0.0031 (4)	0.0070 (5)	−0.0040 (5)
N2A	0.0192 (6)	0.0210 (6)	0.0374 (8)	0.0021 (5)	0.0098 (6)	−0.0096 (6)
C1A	0.0309 (8)	0.0211 (7)	0.0227 (7)	−0.0002 (6)	0.0089 (6)	0.0015 (6)
C2A	0.0364 (8)	0.0294 (8)	0.0220 (7)	0.0000 (7)	0.0127 (6)	−0.0030 (6)
C3A	0.0257 (7)	0.0225 (7)	0.0313 (8)	−0.0003 (6)	0.0140 (6)	−0.0073 (6)
C4A	0.0245 (7)	0.0184 (7)	0.0281 (8)	0.0049 (5)	0.0100 (6)	0.0013 (6)
C5A	0.0232 (7)	0.0197 (7)	0.0211 (7)	0.0037 (5)	0.0085 (5)	0.0004 (5)
C6A	0.0187 (6)	0.0152 (6)	0.0213 (7)	−0.0007 (5)	0.0062 (5)	−0.0021 (5)
C7A	0.0203 (6)	0.0143 (6)	0.0242 (7)	−0.0003 (5)	0.0070 (5)	−0.0004 (5)
C8A	0.0198 (6)	0.0153 (6)	0.0225 (7)	0.0004 (5)	0.0104 (5)	−0.0004 (5)
C9A	0.0681 (14)	0.0369 (10)	0.0309 (9)	0.0167 (10)	0.0169 (9)	0.0128 (8)
S1B	0.01921 (17)	0.02226 (18)	0.02552 (19)	0.00457 (13)	0.00349 (14)	−0.00916 (14)
O1B	0.0238 (5)	0.0242 (5)	0.0222 (5)	0.0056 (4)	0.0017 (4)	−0.0022 (4)
N1B	0.0178 (5)	0.0167 (5)	0.0182 (6)	0.0029 (4)	0.0035 (4)	−0.0036 (4)
N2B	0.0201 (6)	0.0209 (6)	0.0231 (6)	0.0052 (5)	0.0044 (5)	−0.0038 (5)
C1B	0.0230 (6)	0.0196 (7)	0.0142 (6)	−0.0007 (5)	0.0045 (5)	−0.0002 (5)
C2B	0.0297 (7)	0.0183 (7)	0.0200 (7)	−0.0002 (6)	0.0059 (6)	−0.0032 (5)
C3B	0.0304 (8)	0.0240 (7)	0.0248 (7)	0.0073 (6)	0.0068 (6)	−0.0060 (6)
C4B	0.0220 (7)	0.0303 (8)	0.0277 (8)	0.0038 (6)	0.0069 (6)	−0.0079 (6)
C5B	0.0223 (7)	0.0227 (7)	0.0216 (7)	−0.0004 (5)	0.0059 (5)	−0.0056 (6)
C6B	0.0215 (6)	0.0174 (6)	0.0147 (6)	0.0015 (5)	0.0056 (5)	−0.0012 (5)
C7B	0.0207 (6)	0.0171 (6)	0.0179 (6)	−0.0005 (5)	0.0063 (5)	−0.0020 (5)
C8B	0.0188 (6)	0.0155 (6)	0.0195 (6)	0.0015 (5)	0.0083 (5)	−0.0003 (5)
C9B	0.0227 (7)	0.0265 (8)	0.0216 (7)	−0.0043 (6)	0.0063 (6)	−0.0044 (6)

### Geometric parameters (Å, º)

**Table:**

S1A—C8A	1.6858 (15)	S1B—C8B	1.6806 (14)
O1A—C7A	1.2215 (17)	O1B—C7B	1.2207 (17)
N1A—C7A	1.3818 (19)	N1B—C8B	1.3850 (18)
N1A—C8A	1.3845 (18)	N1B—C7B	1.3883 (18)
N1A—H1A	0.80 (2)	N1B—H1B	0.84 (2)
N2A—C8A	1.3083 (18)	N2B—C8B	1.3156 (18)
N2A—H3A	0.83 (2)	N2B—H3B	0.89 (2)
N2A—H2A	0.86 (2)	N2B—H2B	0.83 (2)
C1A—C2A	1.388 (2)	C1B—C2B	1.397 (2)
C1A—C6A	1.403 (2)	C1B—C6B	1.408 (2)
C1A—C9A	1.504 (2)	C1B—C9B	1.507 (2)
C2A—C3A	1.385 (2)	C2B—C3B	1.384 (2)
C2A—H2AA	0.9500	C2B—H2BA	0.9500
C3A—C4A	1.381 (2)	C3B—C4B	1.386 (2)
C3A—H3AA	0.9500	C3B—H3BA	0.9500
C4A—C5A	1.389 (2)	C4B—C5B	1.390 (2)
C4A—H4AA	0.9500	C4B—H4BA	0.9500
C5A—C6A	1.389 (2)	C5B—C6B	1.399 (2)
C5A—H5AA	0.9500	C5B—H5BA	0.9500
C6A—C7A	1.492 (2)	C6B—C7B	1.4909 (19)
C9A—H9AA	0.9800	C9B—H9BA	0.9800
C9A—H9AB	0.9800	C9B—H9BB	0.9800
C9A—H9AC	0.9800	C9B—H9BC	0.9800
			
C7A—N1A—C8A	126.92 (12)	C8B—N1B—C7B	126.81 (12)
C7A—N1A—H1A	115.6 (14)	C8B—N1B—H1B	113.6 (15)
C8A—N1A—H1A	115.7 (14)	C7B—N1B—H1B	119.6 (15)
C8A—N2A—H3A	119.9 (16)	C8B—N2B—H3B	120.3 (14)
C8A—N2A—H2A	118.2 (14)	C8B—N2B—H2B	117.4 (16)
H3A—N2A—H2A	122 (2)	H3B—N2B—H2B	122 (2)
C2A—C1A—C6A	117.74 (14)	C2B—C1B—C6B	117.41 (13)
C2A—C1A—C9A	119.66 (15)	C2B—C1B—C9B	118.81 (13)
C6A—C1A—C9A	122.58 (15)	C6B—C1B—C9B	123.77 (13)
C3A—C2A—C1A	121.71 (15)	C3B—C2B—C1B	121.89 (14)
C3A—C2A—H2AA	119.1	C3B—C2B—H2BA	119.1
C1A—C2A—H2AA	119.1	C1B—C2B—H2BA	119.1
C4A—C3A—C2A	120.05 (14)	C2B—C3B—C4B	120.35 (14)
C4A—C3A—H3AA	120.0	C2B—C3B—H3BA	119.8
C2A—C3A—H3AA	120.0	C4B—C3B—H3BA	119.8
C3A—C4A—C5A	119.48 (14)	C3B—C4B—C5B	119.14 (14)
C3A—C4A—H4AA	120.3	C3B—C4B—H4BA	120.4
C5A—C4A—H4AA	120.3	C5B—C4B—H4BA	120.4
C4A—C5A—C6A	120.29 (14)	C4B—C5B—C6B	120.64 (14)
C4A—C5A—H5AA	119.9	C4B—C5B—H5BA	119.7
C6A—C5A—H5AA	119.9	C6B—C5B—H5BA	119.7
C5A—C6A—C1A	120.71 (13)	C5B—C6B—C1B	120.48 (13)
C5A—C6A—C7A	119.33 (13)	C5B—C6B—C7B	119.09 (13)
C1A—C6A—C7A	119.88 (13)	C1B—C6B—C7B	120.34 (13)
O1A—C7A—N1A	122.93 (13)	O1B—C7B—N1B	122.28 (13)
O1A—C7A—C6A	122.40 (13)	O1B—C7B—C6B	122.72 (13)
N1A—C7A—C6A	114.67 (12)	N1B—C7B—C6B	115.00 (12)
N2A—C8A—N1A	117.98 (13)	N2B—C8B—N1B	118.11 (13)
N2A—C8A—S1A	123.67 (12)	N2B—C8B—S1B	122.68 (11)
N1A—C8A—S1A	118.34 (10)	N1B—C8B—S1B	119.21 (10)
C1A—C9A—H9AA	109.5	C1B—C9B—H9BA	109.5
C1A—C9A—H9AB	109.5	C1B—C9B—H9BB	109.5
H9AA—C9A—H9AB	109.5	H9BA—C9B—H9BB	109.5
C1A—C9A—H9AC	109.5	C1B—C9B—H9BC	109.5
H9AA—C9A—H9AC	109.5	H9BA—C9B—H9BC	109.5
H9AB—C9A—H9AC	109.5	H9BB—C9B—H9BC	109.5
			
C6A—C1A—C2A—C3A	0.1 (2)	C6B—C1B—C2B—C3B	−2.8 (2)
C9A—C1A—C2A—C3A	178.34 (18)	C9B—C1B—C2B—C3B	176.38 (14)
C1A—C2A—C3A—C4A	−0.9 (3)	C1B—C2B—C3B—C4B	0.5 (2)
C2A—C3A—C4A—C5A	0.4 (2)	C2B—C3B—C4B—C5B	1.4 (3)
C3A—C4A—C5A—C6A	1.0 (2)	C3B—C4B—C5B—C6B	−1.0 (2)
C4A—C5A—C6A—C1A	−1.9 (2)	C4B—C5B—C6B—C1B	−1.3 (2)
C4A—C5A—C6A—C7A	174.80 (13)	C4B—C5B—C6B—C7B	−177.71 (14)
C2A—C1A—C6A—C5A	1.3 (2)	C2B—C1B—C6B—C5B	3.2 (2)
C9A—C1A—C6A—C5A	−176.90 (17)	C9B—C1B—C6B—C5B	−175.98 (14)
C2A—C1A—C6A—C7A	−175.35 (14)	C2B—C1B—C6B—C7B	179.53 (13)
C9A—C1A—C6A—C7A	6.5 (2)	C9B—C1B—C6B—C7B	0.4 (2)
C8A—N1A—C7A—O1A	−2.2 (2)	C8B—N1B—C7B—O1B	−0.6 (2)
C8A—N1A—C7A—C6A	178.65 (13)	C8B—N1B—C7B—C6B	178.45 (13)
C5A—C6A—C7A—O1A	−126.72 (16)	C5B—C6B—C7B—O1B	139.95 (15)
C1A—C6A—C7A—O1A	50.0 (2)	C1B—C6B—C7B—O1B	−36.5 (2)
C5A—C6A—C7A—N1A	52.41 (18)	C5B—C6B—C7B—N1B	−39.13 (19)
C1A—C6A—C7A—N1A	−130.91 (15)	C1B—C6B—C7B—N1B	144.47 (13)
C7A—N1A—C8A—N2A	7.1 (2)	C7B—N1B—C8B—N2B	4.7 (2)
C7A—N1A—C8A—S1A	−173.59 (12)	C7B—N1B—C8B—S1B	−176.08 (12)

### Hydrogen-bond geometry (Å, º)

**Table:**

*D*—H···*A*	*D*—H	H···*A*	*D*···*A*	*D*—H···*A*
N1*A*—H1*A*···S1*A*^i^	0.80 (2)	2.60 (2)	3.3227 (16)	151.2 (18)
N1*B*—H1*B*···S1*B*^ii^	0.84 (2)	2.65 (2)	3.4780 (14)	172 (2)
N2*A*—H2*A*···S1*B*^iii^	0.85 (2)	2.49 (2)	3.2945 (15)	157.8 (19)
N2*B*—H2*B*···O1*B*	0.83 (2)	1.98 (2)	2.6404 (18)	136 (2)
N2*A*—H3*A*···O1*A*	0.83 (2)	2.02 (2)	2.6515 (19)	133 (2)
N2*B*—H3*B*···S1*A*^iii^	0.89 (2)	2.49 (2)	3.3800 (14)	177 (2)
C5*B*—H5*BA*···O1*A*^iv^	0.95	2.45	3.3584 (19)	160
C9*B*—H9*BA*···S1*A*^i^	0.98	2.80	3.6946 (17)	152

Symmetry codes: (i) −*x*+1/2, −*y*+1/2, −*z*; (ii) −*x*+1, −*y*+1, −*z*; (iii) −*x*+1/2, −*y*+3/2, −*z*; (iv) *x*+1/2, *y*+1/2, *z*.
