# Supplementary figures and images for: Crystal structure of N-carbamo­thioyl-2-methyl­benzamide
Source: Acta Crystallogr E Crystallogr Commun. 2015 May 28;71(Pt 6):o425. doi: 10.1107/S2056989015009585 (PMC4459304; doi:10.1107/S2056989015009585)

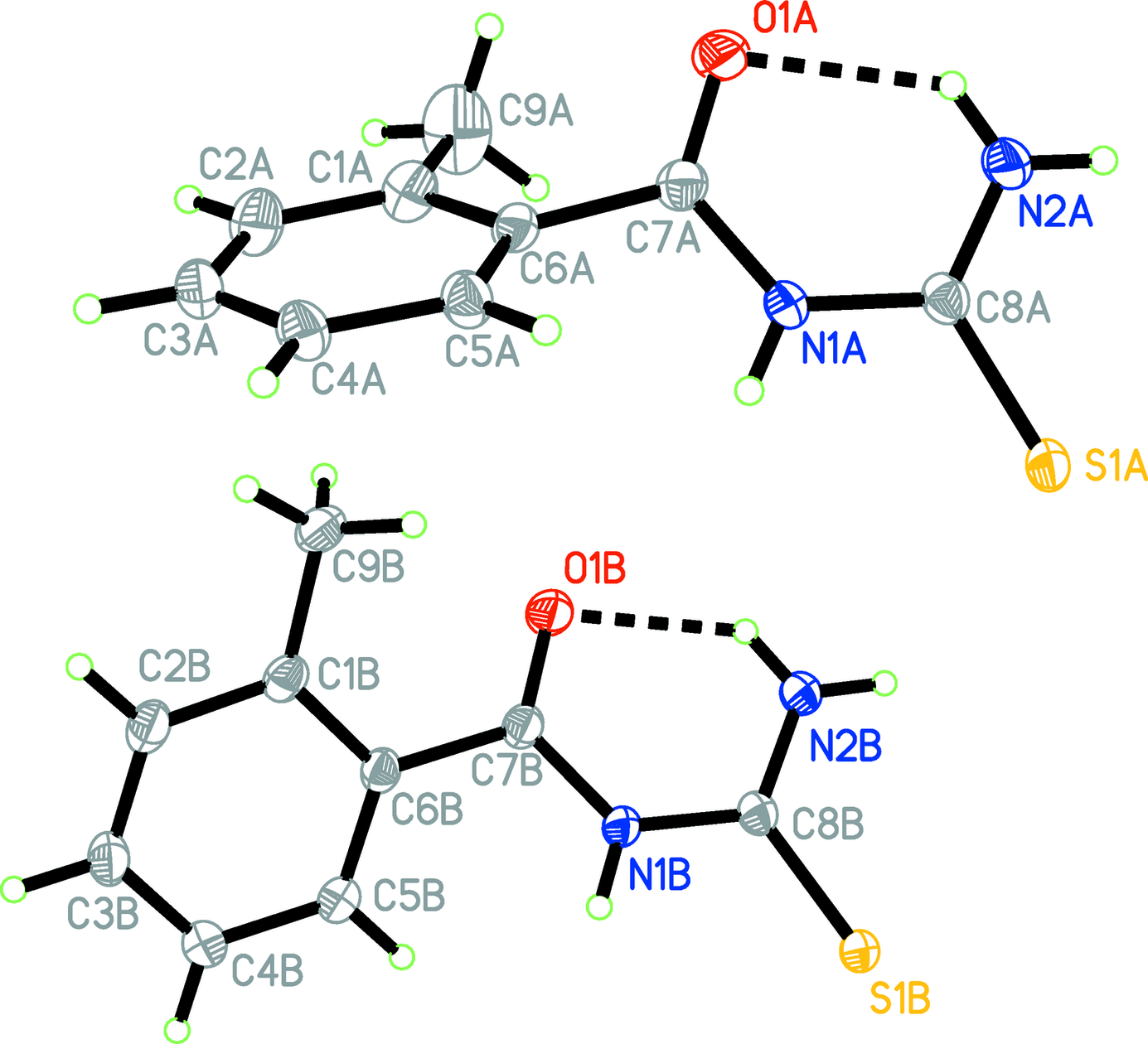

Supplement: Supplementary file 4 [file e-71-0o425-fig1.tif]

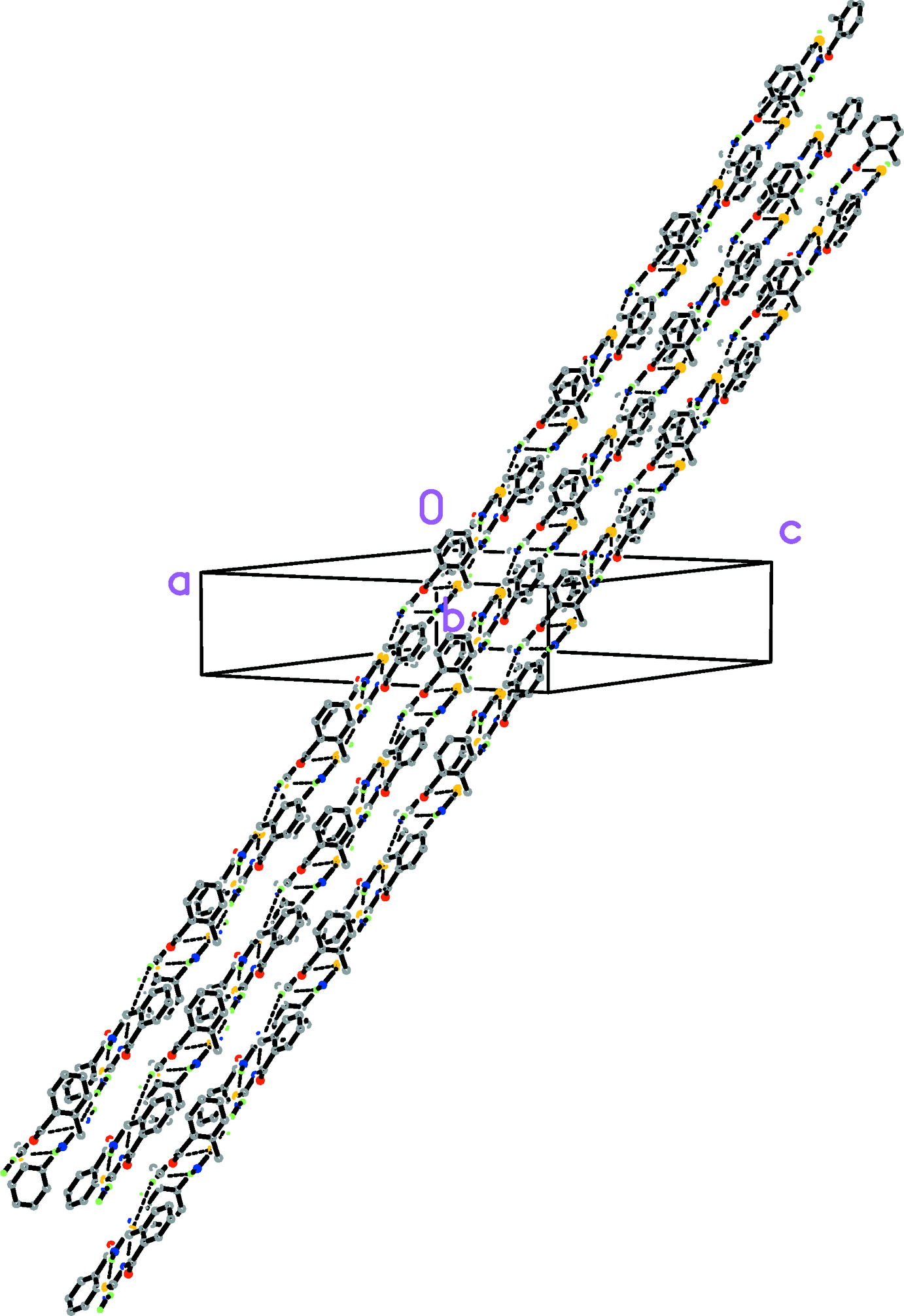

Supplement: Supplementary file 5 [file e-71-0o425-fig2.tif]
